# Feasibility Study of a Novel Protease-Activated Fluorescent Imaging System for Real-Time, Intraoperative Detection of Residual Breast Cancer in Breast Conserving Surgery

**DOI:** 10.1245/s10434-019-08158-1

**Published:** 2020-01-02

**Authors:** Barbara L. Smith, Conor R. Lanahan, Michelle C. Specht, Bridget N. Kelly, Carson Brown, David B. Strasfeld, Jorge M. Ferrer, Upahvan Rai, Rong Tang, Travis Rice-Stitt, Anna Biernacka, Elena F. Brachtel, Michele A. Gadd

**Affiliations:** 1 Division of Surgical Oncology, Massachusetts General Hospital, Boston, MA USA; 2 Department of Pathology, Massachusetts General Hospital, Boston, MA USA; 3 Lumicell, Inc, Newton, MA USA

## Abstract

**Background:**

Obtaining tumor-free margins is critical to prevent recurrence after lumpectomy for breast cancer. Unfortunately, current approaches leave positive margins that require second surgeries in 20–40% of patients. We assessed the LUM Imaging System for real-time, intraoperative detection of residual tumor.

**Methods:**

Breast lumpectomy cavity walls and excised specimens were assessed with the LUM Imaging System after 1 mg/kg intravenous LUM015, a protease-activatable fluorescent agent. Fluorescence at potential sites of residual tumor in lumpectomy cavity walls was evaluated intraoperatively with a sterile hand-held probe, with real-time predictive results displayed on a monitor intraoperatively, and later correlated with histopathology.

**Results:**

In vivo lumpectomy cavities and excised specimens were imaged after LUM015 injection in 45 women undergoing breast cancer surgery. Invasive ductal and lobular cancers and intraductal cancer (DCIS) were included. A total of 570 cavity margin surfaces in 40 patients were used for algorithm development. Image analysis and display took approximately 1 s per 2.6-cm-diameter circular margin surface. All breast cancer subtypes could be distinguished from adjacent normal tissue. For all imaged cavity surfaces, sensitivity for tumor detection was 84%. Among 8 patients with positive margins after standard surgery, sensitivity for residual tumor detection was 100%; 2 of 8 were spared second surgeries because additional tissue was excised at sites of LUM015 signal. Specificity was 73%, with some benign tissues showing elevated fluorescent signal.

**Conclusions:**

The LUM015 agent and LUM Imaging System allow rapid identification of residual tumor in the lumpectomy cavity of breast cancer patients and may reduce rates of positive margins.

## Background

Long-term follow-up of randomized trials has confirmed that breast conserving lumpectomy provides survival equivalent to that of mastectomy for most women with breast cancer.^1^^,^^2^ Current lumpectomy and radiation techniques provide excellent local control, with risk of in-breast recurrence approximately 2–3% for most histological subtypes.^3^ However, preventing in-breast recurrence is important, as it is now recognized that local recurrence can decrease survival, with 1 excess death for every 4 ipsilateral breast tumor recurrences.^4^

Unfortunately, achieving the microscopically tumor-free margins needed to prevent local recurrence is challenging. Current preoperative imaging and surgical techniques still result in positive lumpectomy margins in 20–40% of patients.^5^–^9^ Positive margins require a second surgical procedure to excise additional breast tissue, which increases patient discomfort and anxiety, worsens cosmetic outcomes, and adds to the cost of care.

Most currently available margin assessment tools attempt to identify tumor cells on the surface of an excised lumpectomy specimen.^5^^,^^10^–^15^ Both standard and experimental specimen-based approaches for margin assessment suffer from challenges related to specimen deformation that make orientation unreliable, trauma to specimen surfaces that results in false positive margins, and the inability to assess more than a small fraction of a specimen surface.^16^–^18^

The ideal approach for intraoperative margin assessment for cancer surgery would rapidly identify residual tumor directly in the walls of the surgical cavity, guide additional excision, and verify that clear margins have been achieved. The LUM Imaging System is a cavity-based margin assessment tool that addresses these margin assessment goals. It consists of (1) LUM015, a novel PEGylated protease-activated far-red fluorescent imaging agent;^19^ (2) a hand-held optical head for intraoperative tissue imaging;^20^ and (3) software for image analysis to identify signal associated with residual cancer in the cavity wall.

In a Phase 1 study in 15 human patients, the LUM Imaging System distinguished areas of malignant sarcoma and breast cancer from surrounding normal tissue in ex vivo surgical specimens.^19^ LUM015 was shown to be activated by proteases, particularly cathepsins K, L, S, and B. We recently reported the first human in vivo use of the LUM Imaging System during breast cancer lumpectomy surgery.^21^ Both invasive tumor and ductal carcinoma in situ (DCIS) were rapidly distinguished from normal tissue with high sensitivity and good specificity. We now describe results of a feasibility study of the LUM Imaging System for intraoperative detection of residual tumor during breast cancer lumpectomy surgery.

## Methods

The LUM 2.6 Imaging System (Lumicell, Newton, MA) includes: (1) LUM015, a novel PEGylated protease-activated far-red fluorescent imaging agent;^19^ (2) the LUM 2.6 optical head, a hand-held imaging probe and sterile cover, used to excite LUM015 and collect real-time fluorescent recordings;^20^ and (3) software for image analysis. Use of this system for breast cancer lumpectomies has been previously described.^21^ The imaging probe has a 2.6-cm-diameter circular field of view and an outer diameter of 3.1 cm with an angled neck that facilitates contact with lateral and anterior cavity walls.

We conducted an IRB-approved, prospective, non-randomized, open-label study at Massachusetts General Hospital, Boston, MA. Eligibility included age > 18 years, invasive breast cancer and/or DCIS and a plan for lumpectomy. Patients with prior ipsilateral cancer surgery, open surgical biopsy for diagnosis or neoadjuvant systemic therapy were excluded.

Subjects were injected with 1.0 mg/kg of LUM015 as a 3-min intravenous push, 56–402 min prior to surgery. Marker localization for non-palpable lesions, and sentinel node isotope injection were performed before or after LUM015 injection. Methylene blue and isosulfan blue for node mapping were not used prior to use of the LUM Imaging System.

Surgical procedures were performed by 3 breast surgeons (BLS, MAG, and MCS). After a standard lumpectomy procedure, cavity walls were imaged with the optical head in vivo and fluorescent signal recorded. Shaved margins 0.5–1.0 cm thick were then taken from the entire cavity per standard institutional practice. Cavity walls were imaged again and additional tissue, termed therapeutic shaves, was excised from areas of high fluorescent signal if the LUM Imaging System predicted residual cancer in the cavity. No more than 2 therapeutic shaves, totaling no more than 2 cm thickness, were taken from any given cavity orientation. Excised lumpectomy and shaved margin specimens were imaged ex vivo. During cavity imaging, overhead lights were left on but OR table spotlights were turned off. Breast skin around the incision was covered with a towel while imaging to reduce light exposure. Patients were discharged home the day of surgery and assessed for adverse events at the first postoperative visit.

Tumor:normal (T:N) fluorescent signal ratios were determined by transecting lumpectomy specimens ex vivo and imaging cut surfaces with the LUM System. LUM015 fluorescent signal was correlated with histopathology on hematoxylin and eosin stained slides.^22^

The study started with a tumor detection algorithm established from ex vivo imaging data from our Phase 1^19^ and pilot^21^ studies. This was used to (1) establish the tumor detection threshold for each patient during their intraoperative imaging session and (2) guide the surgeon in excising additional tissue. However, a final detection algorithm was subsequently refined by retrospective analysis of pooled data from all patients. The final detection algorithm was then reapplied to images from the entire population to retrospectively determine what sensitivity and specificity could be achieved with the system.

To create the revised tumor detection algorithm using imaging data from this study, a receiver operating characteristic curve (ROC) was generated after normalizing by the normal-tissue baseline for a given imaging session. A normal-tissue baseline coefficient was varied along the ROC and a point was chosen that optimized both sensitivity and specificity for tumor detection in tissue specimens. Unique thresholds were created for each patient, using the normal-tissue baseline coefficient and the patient’s normal-tissue baseline values. For each image taken, levels of fluorescence exceeding the defined threshold were deemed a “positive” Lumicell image, while levels below threshold were deemed a “negative” Lumicell image.

On histopathology assessment, margins for invasive cancers were considered positive if tumor was present on ink. For DCIS without invasion, margins were positive if DCIS was present < 2 mm from ink. A specimen with a positive Lumicell image was considered a true positive if tumor was found on histopathology examination of the shaved specimen obtained at that site or was a false positive if histopathology found no tumor in that specimen. If no specimen was taken from the cavity wall at the site of the positive Lumicell image, histopathology of the outer surface of the specimen excised from that cavity orientation was used to determine margin status.

## Results

### Patient and Tumor Characteristics

Forty-five breast cancer patients received intravenous LUM015 at 1.0 mg/kg 56–402 min prior to surgery. Median age was 59 years (range 44–79) with 14 (31%) premenopausal or perimenopausal and 31 (69%) postmenopausal. Thirty-five patients required wire or seed localization for excision of non-palpable tumors. Mean tumor size was 1.2 cm (range 0.06–3.5 cm). Tumor histology included 25 (55%) invasive ductal carcinomas ± DCIS, 5 (11%) invasive lobular carcinomas, 3 (7%) invasive carcinomas with ductal and lobular features, and 12 (27%) DCIS only (Table 1). Breast density on mammography was heterogeneously or extremely dense in 24 (54%), fatty or scattered fibroglandular densities in 19 (42%) and mixed dense and scattered density in 2 (4%).

**Table 1:** Patient demographics

Number of patients	45
Median age (range)	59 years (44-79)
Mean BMI (kg/m^2^) (range)	27.6 ± 5.6 (20.4-44.4)
Ethnicity	
White	38 (84%)
Black	5 (11%)
Asian	2 (5%)
Menopausal status	
Post	31 (69%)
Pre/Peri	14 (31%)
Mammographic breast density	
Almost entirely fatty	1 (2%)
Scattered areas of fibroglandular density	18 (40%)
Heterogeneously dense	22 (49%)
Extremely dense	2 (4.5%)
Mixed scattered fibroglandular/heterogeneously dense	2 (4.5%)
Physical examination findings	
Palpable mass	13 (29%)
No palpable mass	32 (71%)
Tumor histology (biopsy and/or main lumpectomy specimen)	
Invasive ductal carcinoma ± DCIS	25 (55%)
Invasive lobular carcinoma	5 (11%)
Invasive carcinoma with ductal and lobular features	3 (7%)
DCIS only	12 (27%)

### Establishment of Imaging Protocols

The minimum acceptable timepoint to begin imaging after injection of LUM015 was determined by finding the shortest timepoint at which the detection algorithm successfully predicted the presence of tumor. The lower bound for the imaging timepoint was 101 min. Two patients were excluded from the analysis because the imaging timepoint was less than 101 min (56 and 92 min). The longest time between injection and imaging in the study was 402 min, and the signal remained acceptable at this timepoint.

After excision of the main lumpectomy specimen, cavity wall scanning to initialize the image analysis software took approximately 30 s. The surgeon then scanned the entire cavity with the imaging probe, viewing a 2.6-cm-diameter section of cavity wall image displayed on a monitor. The software applied red color to areas where the fluorescent signal was above the threshold indicating possible residual tumor, and displayed areas below signal in gray-scale. Image acquisition was nearly instantaneous, with red color displayed in real time as the probe was moved along cavity walls. Imaging of the entire cavity could be completed in 1–2 min for most patients. For patients where additional tissue was excised at areas of high LUM015 signal, repeat scanning required approximately another minute.

Five patients’ data were not used in development of the tumor detection algorithm. In 2 patients, cavities were imaged at less than the 101-min minimum interval between LUM015 injection and imaging; 1 patient did not receive the planned LUM015 dose due to extravasation of LUM015 into surrounding tissue during injection; 1 tumor cavity had extensive necrotic tissue and hematoma at the core biopsy site with limited fluorescent signal; and 1 patient’s intraoperative imaging data was lost before analysis. This left 40 patients whose data was used for algorithm development. Safety assessments were performed on all 45 patients who received LUM015 injections.

In some cases, probe contact with lumpectomy cavity walls was adversely affected by incisions that were small (< 3 cm) relative to probe size, by suboptimal probe positioning by the surgeon, or by other technical factors. We assumed that the signal in vivo should be equal to or greater than the signal from an ex vivo shaved cavity margin specimen from that site. The in vivo cavity wall signal includes total tissue signal. Signal from the ex vivo shaved margin specimen could contain all or part of the total tissue signal from that site but should not contain a higher signal. Thus, we expected that the in vivo/ex vivo signal ratio would be approximately 1 or greater when there was good tissue contact between the probe and cavity wall. Ratios less than 1 would suggest poor in vivo probe contact and poor signal data. Our analysis indicated that ratios of less than or equal to 0.84 suggested poor contact between the imaging probe and the cavity walls. We excluded 8 cavity images with contact scores below 0.84.

### System Performance

**Fig. 1:**
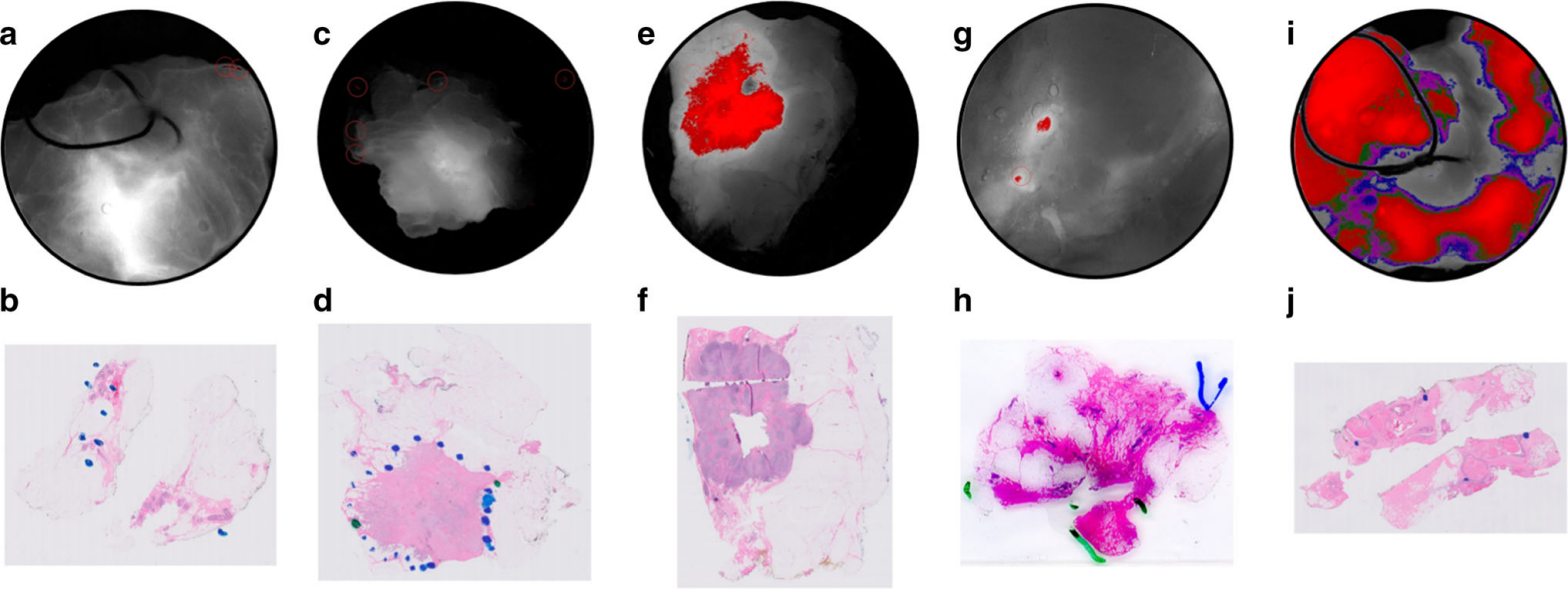
**a**–**b** Ex vivo LUM Image of the marginal aspect of a comprehensive shaved cavity margin*; Perpendicularly sectioned multifocal, grade 2 ductal carcinoma in situ (DCIS) with necrosis, calcifications and cancerization of lobules, < 1 mm to inked margin. **c**–**d** Ex vivo LUM Image of lumpectomy cross section*; grade 1 invasive ductal carcinoma (IDC), 0.9 cm across. **e**–**f** Ex vivo LUM Image of lumpectomy cross section*; grade 3 IDC with extensive intraductal component and prominent lymphocytic infiltrate, 1.3 cm across. **g**–**h** In vivo LUM Image of final margin with red highlighting for signal above threshold; grade 1 to 2 invasive lobular carcinoma, extensive lobular carcinoma in situ, focal atypical ductal hyperplasia, fibrocystic changes with usual ductal hyperplasia and apocrine cysts, 0.6 cm present at the inked margin along a broad front. **i**–**j** Ex vivo LUM Image of the marginal aspect of a comprehensive shaved cavity margin*; Perpendicularly sectioned grade 2 to 3 DCIS with associated necrosis and calcifications spanning up to 0.7 cm, present or < 0.1 cm from new inked margin along a broad front. *The LUM Imaging System algorithm for signal-highlighting produces red highlights during in vivo imaging. Some ex vivo images are displayed as black and white images without red highlights

A total of 570 cavity margin surfaces in 40 patients were imaged intraoperatively and compared to excised specimen histopathology to develop the tumor detection algorithm. Image acquisition for each 2.6-cm-diameter margin surface took approximately 1 s. Invasive ductal carcinoma, invasive lobular carcinoma, invasive carcinoma with mixed ductal and lobular features and DCIS all produced similar fluorescent signals, with tumor fluorescent signal 3.78–4.11 times greater than normal tissue signal (Fig. 1). Tumor could be distinguished from normal tissue in ex vivo transected lumpectomy specimens regardless of breast mammographic density or patient menopausal status.

### Development and Refinement of the Tumor Detection Algorithm

Fluorescent signal measured in excised specimens and lumpectomy cavity walls was used to develop the tumor detection algorithm. After excision of the standard-of-care main lumpectomy specimen, the cavity was scanned with the optical head and a normal-tissue baseline was determined. A unique tumor signal threshold was created for that patient. During cavity scans, the software applied the detection algorithm to the acquired signal and produced images with red-colored highlights on the monitor, indicating areas of high fluorescent signal suspicious for tumor.

Imaging data from the lumpectomy cavity walls of all patients was used to create a final tumor detection algorithm. A receiver operating characteristic curve (ROC) was generated after normalizing by the normal-tissue baseline for a given imaging session. A normal-tissue baseline coefficient was varied along the ROC and a point was chosen that optimized both sensitivity and specificity for tumor detection in tissue specimens. Normal tissue was defined as imaged tissue that contained no tumor on final pathology assessment.

The final tumor detection algorithm was applied to all 570 margin images, including 257 intermediate cavity surfaces [cavity images acquired after removal of main specimen but before taking comprehensive shaved cavity margins (SCM)] and 313 final cavity surfaces (images acquired after comprehensive SCM were taken). Assessment of LUM015 signal was performed on a per margin basis, with a margin orientation (e.g., superior) deemed positive if there was any area of LUM015 signal above threshold. For larger cavities, several 2.6-cm probe field-of-view areas would be combined for complete assessment of a margin surface. Similarly, a margin orientation was deemed pathology positive if tumor was present anywhere in that margin.

There were 17 surfaces that contained tumor on standard histopathology, 9 intermediate margins and 8 final margins. Tumor signal was below threshold in 3 of 17 images, all of which were intermediate cavity surfaces. For all cavity surfaces, sensitivity for tumor detection was 84%. Specificity was 73%, with some benign tissues showing elevated fluorescent signal. For intermediate margins, device readings were correlated with pathology findings in tissue excised from that site (Table 2). For the 8 patients with positive final margins after excision of the main lumpectomy specimen and standard-of-care comprehensive shaved margins, sensitivity for detection of residual tumor in the final cavity margin was 100% (Table 3).

**Table 2:** LUM Imaging System readings at 9 histopathology positive intermediate lumpectomy margins

Histology of shaved cavity margin	LUM Imaging System reading of in vivo cavity wall at that site
IC on ink	+
IDC on ink	+
IDC < 1 mm from ink	+
IDC < 1 mm from ink, DCIS on ink	+
IDC > 2 mm from ink	+
IDC > 2 mm from ink	–
DCIS < 2 mm from ink	+
DCIS < 2 mm from ink	–
DCIS > 2 mm from ink	–

IC, invasive carcinoma with ductal and lobular features; IDC, invasive ductal cancer; DCIS, ductal carcinoma in situ

**Table 3:** LUM Imaging System versus standard histopathology assessment of positive final lumpectomy margins

Histopathology at final surface of excised standard-of-care specimen *N* = 8	In vivo LUM Imaging System reading of corresponding cavity wall	Tumor found in additional cavity wall tissue taken at that site	Histology
DCIS < 2 mm from ink	+	+	DCIS
DCIS < 2 mm from ink	+	–	Benign
DCIS < 2 mm from ink	+	+	DCIS^a^
IDC on ink	+	+	IDC^a^
ILC on ink	+	+	ILC^a^
DCIS < 2 mm from ink	+	–	Benign^a^
IDC on ink	–	–	Benign^a^
DCIS < 2 mm from ink	–	–	Benign^a^

DCIS, ductal carcinoma in situ; IDC, invasive ductal cancer; ILC, invasive lobular cancer

^a^Surgeon did not excise additional margin during the initial procedure. Pathology results are from re-excision procedure performed at a later date

### Clinical Outcomes

Two of 8 patients (25%) with positive margins after standard of care surgery were spared second surgeries because additional tissue was excised at sites of high LUM015 signal. The LUM Imaging System correctly predicted that no additional tumor would be found at re-excision in 2 other patients (25%) with positive margins. In the remaining 4 patients with positive lumpectomy margins, the surgeon elected not to take additional tissue at sites of high LUM015 signal. These 4 patients all required second surgical procedures.

No patient had a serious adverse event related to trial participation. One patient had extravasation of LUM015 during preoperative IV injection resulting in blue discoloration of antecubital skin. This resolved without intervention over 3 months. One patient had a hematoma at her lumpectomy site, possibly related to use of the device, which resolved without intervention. One patient had perioperative nausea, and another had transient perioperative hypertension, both unlikely to be related to trial participation.

## Discussion

Obtaining tumor-free lumpectomy margins during breast conserving surgery remains challenging.^5^–^9^ Better approaches for real-time, intraoperative margin assessment are needed.

Our study’s cavity-based margin assessment system has the advantage of identifying the location of residual tumor in the lumpectomy cavity wall. In contrast, specimen-based margin assessment approaches identify tumor on excised specimens, but do not precisely identify the corresponding location of residual tumor in the cavity. Importantly, our approach allows for immediate identification and excision of residual tumor, and allows repeat imaging to verify that the entire suspicious area has been removed.

The LUM Imaging System’s speed of image acquisition and large field of view allowed rapid assessment of the entire lumpectomy cavity, taking only 1 s to image each 2.6-cm-diameter area of cavity wall. By comparison, other clinical and experimental devices for margin assessment have the limitation of only 0.5- to 1.0-cm-diameter fields of view.^12^–^15^

The LUM Imaging System performed well across a wide range of breast cancer types and patient characteristics. Invasive ductal cancers, invasive lobular cancers, and ductal carcinoma in situ could all be distinguished from surrounding normal tissue. LUM015 produced good tumor-to-normal signal ratios in both premenopausal and postmenopausal patients, and in women with high and low breast density as measured by mammography.

Generation of sufficient fluorescence for tumor detection required at least 100 min between LUM015 injection and lumpectomy cavity imaging. Intervals of up to 6 h worked well, with no maximum acceptable interval between injection and imaging yet established. Tumor autofluorescence has been previously documented,^23^^,^^24^ and did not impact performance of the LUM Imaging System in distinguishing tumor from benign tissue.

We correlated fluorescent signal in lumpectomy cavity walls and excised specimens with histopathology to develop algorithms for distinguishing tumor from normal tissue. The algorithm was designed to maximize sensitivity for identifying residual tumor, to reduce rates of re-excision surgery. We accepted that this could increase false positive readings, and increase excised tissue volume in some patients. We believed that a single, slightly larger lumpectomy would have a better cosmetic outcome than that achieved after a second surgery, and would avoid the extra pain, anxiety, and cost associated with second surgeries.

The algorithm developed yielded 84% sensitivity for tumor detection among all eligible imaged surfaces, and 100% sensitivity in the final cavity margin. In this feasibility study, we speculate that surgeons may have spent less time scanning intermediate margins where they knew additional tissue would be excised from the cavity, and more time scanning the final margin where use of the device would impact actions taken. We are working to standardize scanning procedures to reduce false negative readings in future studies.

Not all areas of LUM015 signal were excised by participating surgeons in this feasibility study. In some cases, the surgeon was comfortable using the device for data collection on intermediate margins but was reluctant to take additional tissue from the final cavity wall early in algorithm development. Even so, 25% of patients with positive margins were spared a second surgery as a result of additional tissue excision based on LUM015 signal.

Several factors may have contributed to creating false positive readings. The LUM015 fluorescent signal is produced by proteases around tumors, so that fluorescent signal extends beyond the tumor border. Although this aspect of the system may result in some false positive cavity readings with excision of additional margin tissue, it may help achieve the 1–2 mm of clear margin required for DCIS and desirable for invasive tumor.^25^ Technical factors related to specimen processing and tissue sampling can also affect rates of false positive margins, as only a few 5-μm-thick sections are examined per margin specimen. In some cases, tumor might have been present in excised tissue but not on the slides examined. No specific histological finding consistently caused false positive signals. Work is under way to identify causes of false positive signal and allow development of more specific tumor detection algorithms.

Several other factors affected system performance. Poor contact between the optical head and lumpectomy cavity wall reduced detection of fluorescent signal and may have caused false negative readings. We adjusted intraoperative protocols to verify optical head contact with the cavity wall and developed a contact score to confirm contact intraoperatively. In addition, a smaller optical head has been designed to improve contact and maneuverability in small incisions.

Tissue trauma from core needle biopsies, radioisotope injection for node mapping, and marker placement for non-palpable tumors did not affect detection of fluorescent signal. However, no LUM015 fluorescence was detected in a patient with a significant core biopsy site hematoma with necrotic tissue. We now exclude patients with large hematomas or those with open surgical biopsy for diagnosis.

No study patient had a serious adverse event attributed to LUM015 injection or use of the Lumicell optical head. One patient in a separate trial had an allergic reaction after LUM015 injection but recovered completely (personal communication).

We are currently conducting multicenter trials of the LUM Imaging System in breast cancer lumpectomy surgery (NCT03321929) and are testing and will refine tumor detection algorithms across different breast cancer histological subtypes and after neoadjuvant therapy. The LUM Imaging System is also being evaluated in clinical trials for peritoneal (NCT03834272), central nervous system (NCT03717142), and prostate (NCT03441464) cancers, and is being assessed for use in ovarian, esophagus, pancreas, and colorectal cancers.^26^^,^^27^
